# Characterization of traumatized muscle-derived multipotent progenitor cells from low-energy trauma

**DOI:** 10.1186/s13287-020-02038-2

**Published:** 2021-01-06

**Authors:** Marvin Dingle, Stephen D. Fernicola, Jaira F. de Vasconcellos, Sonia Zicari, Christopher Daniels, John C. Dunn, Alexander Dimtchev, Leon J. Nesti

**Affiliations:** 1 Clinical and Experimental Orthopaedics, Department of Surgery, Uniformed Services University of the Health Sciences, 4301 Jones Bridge Road, Bethesda, MD 20814 USA; 2 Department of Orthopaedic Surgery, Walter Reed National Military Medical Center, 4801 Rockville Pike, Bethesda, MD 20889 USA; 3 Henry M. Jackson Foundation for the Advancement of Military Medicine, 6720A Rockledge Drive, Bethesda, MD 20817 USA; 4 William Beaumont Army Medical Center, 5005 N Piedras St, El Paso, TX 79920 USA

**Keywords:** Stem cell, Multipotent progenitor cells, Wound healing, Low-energy fractures

## Abstract

**Background:**

Multipotent progenitor cells have been harvested from different human tissues, including the bone marrow, adipose tissue, and umbilical cord blood. Previously, we identified a population of mesenchymal progenitor cells (MPCs) isolated from the traumatized muscle of patients undergoing reconstructive surgery following a war-related blast injury. These cells demonstrated the ability to differentiate into multiple mesenchymal lineages. While distal radius fractures from a civilian setting have a much lower injury mechanism (low-energy trauma), we hypothesized that debrided traumatized muscle near the fracture site would contain multipotent progenitor cells with the ability to differentiate and regenerate the injured tissue.

**Methods:**

The traumatized muscle was debrided from the pronator quadratus in patients undergoing open reduction and internal fixation for a distal radius fracture at the Walter Reed National Military Medical Center. Using a previously described protocol for the isolation of MPCs from war-related extremity injuries, cells were harvested from the low-energy traumatized muscle samples and expanded in culture. Isolated cells were characterized by flow cytometry and q-RT-PCRs and induced to adipogenic, osteogenic, and chondrogenic differentiation. Downstream analyses consisted of lineage-specific staining and q-RT-PCR.

**Results:**

Cells isolated from low-energy traumatized muscle samples were CD73+, CD90+, and CD105+ that are the characteristic of adult human mesenchymal stem cells. These cells expressed high levels of the stem cell markers *OCT4* and *NANOG* 1-day after isolation, which was dramatically reduced over-time in monolayer culture. Following induction, lineage-specific markers were demonstrated by each specific staining and confirmed by gene expression analysis, demonstrating the ability of these cells to differentiate into adipogenic, osteogenic, and chondrogenic lineages.

**Conclusions:**

Adult multipotent progenitor cells are an essential component for the success of regenerative medicine efforts. While MPCs have been isolated and characterized from severely traumatized muscle from high-energy injuries, here, we report that cells with similar characteristics and multipotential capacity have been isolated from the tissue that was exposed to low-energy, community trauma.

## Background

Low-energy trauma is among the largest events that lead to bone fracture. Epidemiological studies have shown that the distal radius is among the most frequent site for low-energy fractures in the pediatric and elderly population [1–4]. Distal radius fracture risk factors include age, gender, health condition, and environmental factors, such as climate [5]. Low-energy fractures can be challenging to treat due to a low healing potential and, in many cases, weakened bone strength. Treatment strategies will vary depending on the complexity of the fracture, including the use of open reduction and internal fixation procedure that provides the stability needed to maintain an appropriate alignment and improve biomechanical performance during early rehabilitation [6, 7].

Multipotent progenitor cells have been harvested from many different human tissues, including the bone marrow, trabecular bone, dental pulp, adipose tissue, umbilical cord blood, and high-energy war-traumatized muscle [8–14]. Previously, we identified a population of mesenchymal progenitor cells (MPCs) isolated from the traumatized muscle of patients undergoing reconstructive surgery following war-related blast injuries [12]. These cells demonstrated the ability to differentiate into multiple mesenchymal lineages (adipogenic, osteogenic, and chondrogenic) and expressed a molecular phenotype characteristic of each lineage following induction. More recently, MPC-secreted factors were shown to be sufficient to improve axon growth and cell migration in vitro and MPCs’ neurotrophic activity was enhanced by in vitro biological induction via VEGF-A production, conditioned medium combination with endothelial cells, and/or the co-culture with adult endothelial cells [15, 16]. Altogether, these findings demonstrate the multipotential capacity and neurotrophic activities of human MPCs, and their potential to be further developed as cellular therapies to promote tissue and/or peripheral nerve regeneration.

The high-energy transmitted to the soft tissues from the blast injury is absorbed as thermal, mechanical, and chemical stress triggering multiple cell- and tissue-specific events following injury, such as cell necrosis, apoptosis, and inflammation. While fractures from an urban/civilian setting have a lower injury mechanism (low-energy trauma) and a less robust inflammatory response than high-energy blast injuries, we hypothesized that debrided traumatized muscle near the fracture site in low-energy injuries contains multipotent progenitor cells with the ability to differentiate into multiple mesenchymal lineages in vitro and the potential to be involved and used in tissue regeneration applications.

## Methods

### Ethics statement and clinical samples

Tissue samples were taken from injured patients (6 males and 5 females) undergoing open reduction and internal fixation for an isolated distal radius fracture (*n* = 10) and a humerus fracture (*n* = 1) from a low-energy injury mechanism at the Walter Reed National Military Medical Center. For the purpose of this study, the low-energy injury was defined as non-wartime blunt trauma. This type of injury, in this context, was defined as the absence of traumatic cardiac arrest during transport; systolic blood pressure less than 90; respiratory compromise/intubation; Glasgow coma scale less than 8; traumatic limb paralysis; amputation proximal to wrist or ankle; vascular compromise of extremity; burns with traumatic component; penetrating injuries to head, neck chest, abdomen, extremities proximal to the elbow or knee; vital sign instability; flail chest or multiple rib fractures; pneumothorax/hemothorax; open/depressed skull fractures; two or more long bone fractures; crush injury to chest or pelvis; and high level of suspicion related to the mechanism. The average patient age from which surgical specimens were obtained was 33.5 years old (range 18 to 63 years old), and the average time between injury and the surgical procedure was 10.2 days (range 4 to 24 days). Tissue specimens used in this study were taken at the margin of devitalized and healthy appearing tissue that would otherwise be discarded as surgical waste. The Walter Reed National Military Medical Center Institutional Review Board approved this tissue procurement protocol and waived the need for consent. Typically, the injured muscle tissue at the site of the fracture is debrided in order to facilitate the reduction of the fracture and placement of internal fixation. The amount of tissue debrided was determined by the operative surgeon. After surgical debridement of the tissue, de-identified samples were placed in a sterile container and transported on ice to the laboratory for processing.

### Cell harvest and culture

Cells were harvested from the traumatized muscle tissue of patients undergoing surgical debridements from low-energy injuries as previously described [12, 17]. Debrided tissue, from the traumatized pronator quadratus muscle at the site of injury, was removed in the usual fashion to facilitate the reduction of the fracture and placement of internal fixation. Briefly, the healthy margin of the debrided tissue was washed in Hank’s Balanced Salt Solution (HBSS, Gibco, Carlsbad, CA), minced, and incubated with Collagenase type 2 [0.5 mg/mL] (Worthington Biochemical, Lakewood, NJ) for 2 h at 37 °C with agitation. Following incubation, the tissue was filtered through cell strainers (100 μM and 40 μM, Falcon/Corning, Corning, NY), pelleted by centrifugation, resuspended in growth medium [Dulbecco’s Modified Eagle’s Medium (Gibco) supplemented with 10% fetal bovine serum (FBS, Gibco) and 3X Penicillin/Streptomycin and Fungizone (Gibco)]. Cells were plated and incubated at 37 °C on tissue culture plastic for 2 h and then washed with HBSS to remove the non-adherent cells and enrich mesenchymal progenitor cell (MPC) isolation. After 1 day in culture, growth medium consisted of Dulbecco’s Modified Eagle’s Medium (Gibco) supplemented with 10% FBS (Gibco) and 1X Penicillin/Streptomycin and Fungizone (Gibco). The culture of the adherent cells was maintained until confluence (approximately 2 weeks), and cells were used for differentiation induction experiments between passages 3 and 6.

### Flow cytometry analyses

Cell surface makers were investigated following standard immunophenotyping protocols and as previously described [12, 17] using anti-human CD73-PE Clone AD2, CD90-FITC Clone 5E10, CD105-APC Clone 43A3, CD11b-BV421 Clone ICRF44, and CD45-APC-Cy7 Clone 2D1 antibodies (BioLegend, San Diego, CA). Isolated mesenchymal progenitor cells (MPCs) between passages 2 and 4 were used for these studies. Compensation was performed with compensation beads following manufacture’s protocols (BD CompBeads, Cat #552843, BD Biosciences, San Jose, CA). Fluorescence was analyzed in a BD LSRII flow cytometer (BD Biosciences) at the Flow Cytometry Core, Biomedical Instrumentation Center, Uniformed Services University of the Health Sciences.

### Adipogenesis, osteogenesis, and chondrogenesis differentiation

Cells isolated from the low-energy traumatized human muscle tissue were induced to adipogenic, osteogenic, and chondrogenic differentiation as previously described [12, 17]. For adipogenic differentiation, monolayer cultures of multiprogenitor cells were seeded at a density of 40,000 cells/cm^2^ and treated for 4 weeks with adipogenic medium, consisting of Dulbecco’s modified Eagle’s medium with 10% FBS supplemented with 0.5 mM 3-isobutyl-1-methylxanthine (Acros Organics, Geel, Belgium), 1 μM dexamethasone, and 1 μg/mL insulin (both from Sigma-Aldrich, St. Louis, MI). For osteogenic differentiation, monolayer cultures of multiprogenitor cells were seeded at a density of 5000 cells/cm^2^ and treated for 4 weeks with osteogenic medium, consisting of Dulbecco’s modified Eagle’s medium with 10% FBS supplemented with 10 mM β-glycerol phosphate (Sigma-Aldrich), 50 μg/mL ascorbic acid (Sigma-Aldrich), 10 nM 1,25-di-hydroxyvitamin D_3_ (BIOMOL International, Plymouth Meeting, PA), and 0.01 μM dexamethasone (Sigma-Aldrich). Finally, chondrogenic differentiation was performed in high-density pellets culture (2.5 × 10^5^ cells per pellet) and treated for 4 weeks with chondrogenic medium, consisting of Dulbecco’s modified Eagle’s medium supplemented with 1% insulin, human transferrin, and sodium selenite (ITS) Liquid Media Supplement (Sigma-Aldrich); 10 ng/mL transforming growth factor-β (Sigma-Aldrich); and 0.1 mM dexamethasone (Sigma-Aldrich). For comparison (control), cells were cultured in growth medium consisted of Dulbecco’s modified Eagle’s medium supplemented with 10% fetal bovine serum and 1X Penicillin/Streptomycin and Fungizone (Gibco). After inductions, monolayered cultured cells were fixed and stained with Oil Red O solution (Sigma-Aldrich) for intracellular lipid droplets (adipogenic differentiation) or with 2% Alizarin Red S at pH 4.2 (Sigma-Aldrich) for evidence of a mineralized matrix (osteogenic differentiation) as previously described [17]. Chondrogenic pellets were fixed after 4 weeks, dehydrated, embedded in paraffin, sectioned [5-μM thickness] and stained with Alcian blue staining solution (EMD Millipore, Temecula, CA) for sulfated glycosaminoglycans as previously described [12].

### RNA isolation and quantitative PCR analysis

Gene expression analyses for *OCT4* and *NANOG* genes were performed on the total pool of traumatized muscle cells at the isolation day (total cells) and following cells isolation on days 1, 3, 5, and 7. Gene expression analyses for adipogenic (*FABP4*, *LPL*, and *PPAR-gamma2*), osteogenic (*ALP*, *CBFA1*, and *Osteocalcin*), and chondrogenic genes (*Aggrecan*, *SOX9*, and *COL2A1*) were performed following each respective differentiation induction. The list of primers used in this study can be found on Supplemental Table 1. RNA was extracted using TRIzol (Thermo Fisher Scientific/Invitrogen, Carlsbad, CA) following manufacturer’s instructions and purified using RNeasy Mini-columns (Qiagen, Germantown, MD). RNA concentration was measured with a Nanodrop spectrophotometer (ThermoFisher Scientific), where RNA quality corresponded to an A260/280 value of at least 1.8 followed by cDNA synthesis. Relative gene expression analyses were performed by q-RT-PCR with an Applied Biosystems QuantStudio 7 Flex real-time PCR detection system (Applied Biosystems, Foster City, CA). Gene expression was normalized using *GAPDH* or *HPRT1* as an internal housekeeping control.

### Statistical analysis

Replicates are expressed as mean + standard error values and significance was calculated by two-tailed Student’s *t* test.

## Results

### Human cells isolated from low-energy traumatized muscle samples express CD73, CD90, and CD105 surface markers

**Fig. 1:**
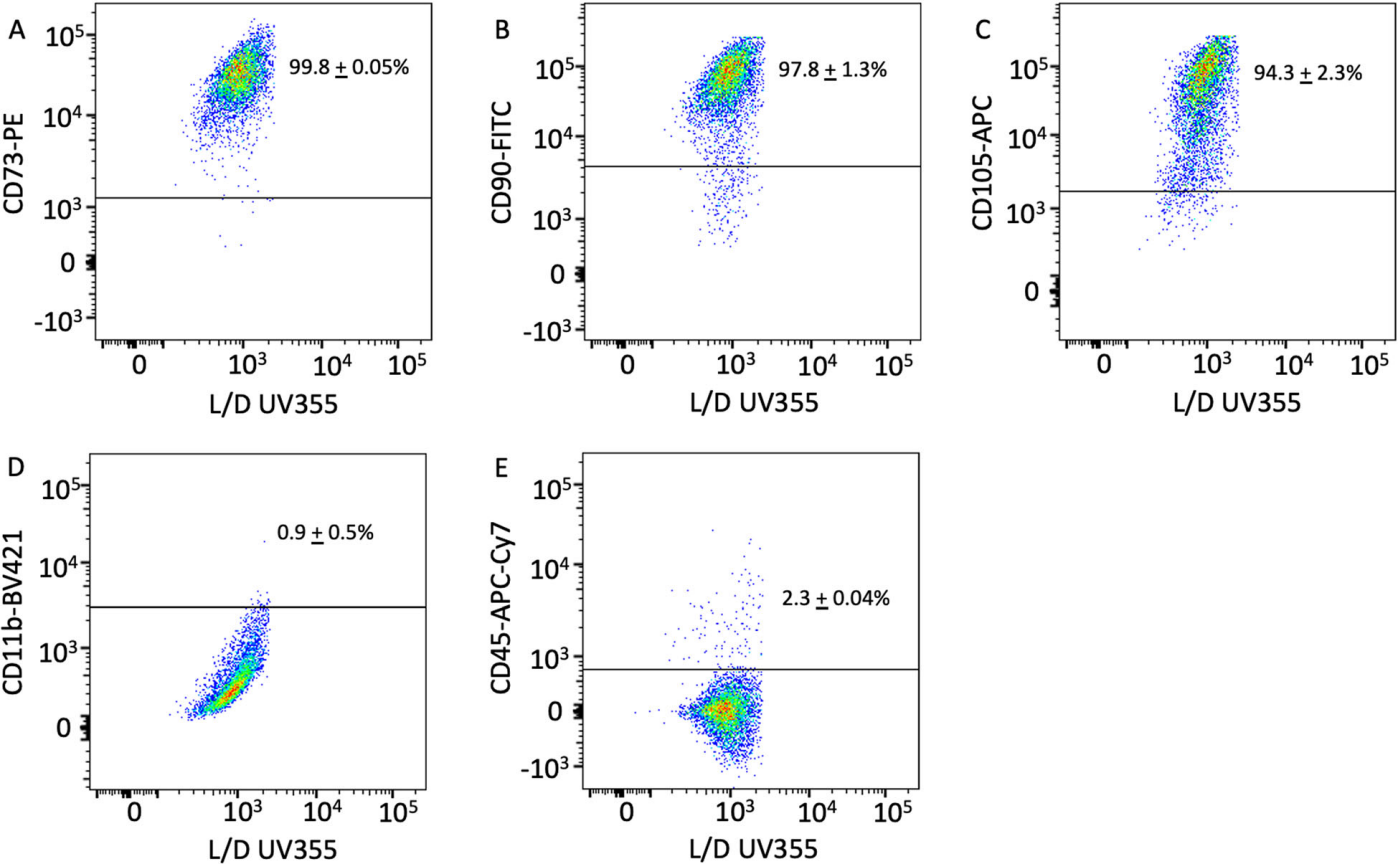
Cells isolated from the low-energy traumatized muscle samples express CD73, CD90, and CD105 surface markers. Representative flow dot plots of cells isolated from the low-energy traumatized tissue and stained with **a** CD73, **b** CD90, **c** CD105, **d** CD11b, and **e** CD45. Cells were identified and gated on the basis of their size (forward scatter) and granularity (side scatter), followed by analysis of cells stained with each respective antibody. Mean value + standard error of two independent donors for each staining

We have previously identified and characterized a population of human mesenchymal progenitor cells (MPCs) isolated from the traumatized muscle of patients undergoing reconstructive surgery following war-related blast injuries [12]. These cells also demonstrated the ability to differentiate into multiple mesenchymal lineages [12, 17]. To determine if the cells isolated from low-energy trauma have similar characteristics to the previously reported MPCs isolated from high-energy trauma [12], we performed flow cytometry analysis on the cells isolated from low-energy traumas. As shown on Fig. 1a–c, low-energy trauma cells express the cell surface markers CD73 (average + standard error: 99.8 + 0.05%), CD90 (average + standard error: 97.8 + 1.3%), and CD105 (average + standard error: 94.3 + 2.3%), which are the characteristic of adult human mesenchymal stem cells and high-energy trauma MPCs [12]. In addition, low-energy trauma cells are negative for CD11b (average + standard error: 0.9 + 0.5%) and CD45 (average + standard error: 2.3 + 0.04%), which are the characteristic of monocytes/macrophages and leukocytes, respectively (Fig. 1d, e).

**Fig. 2:**
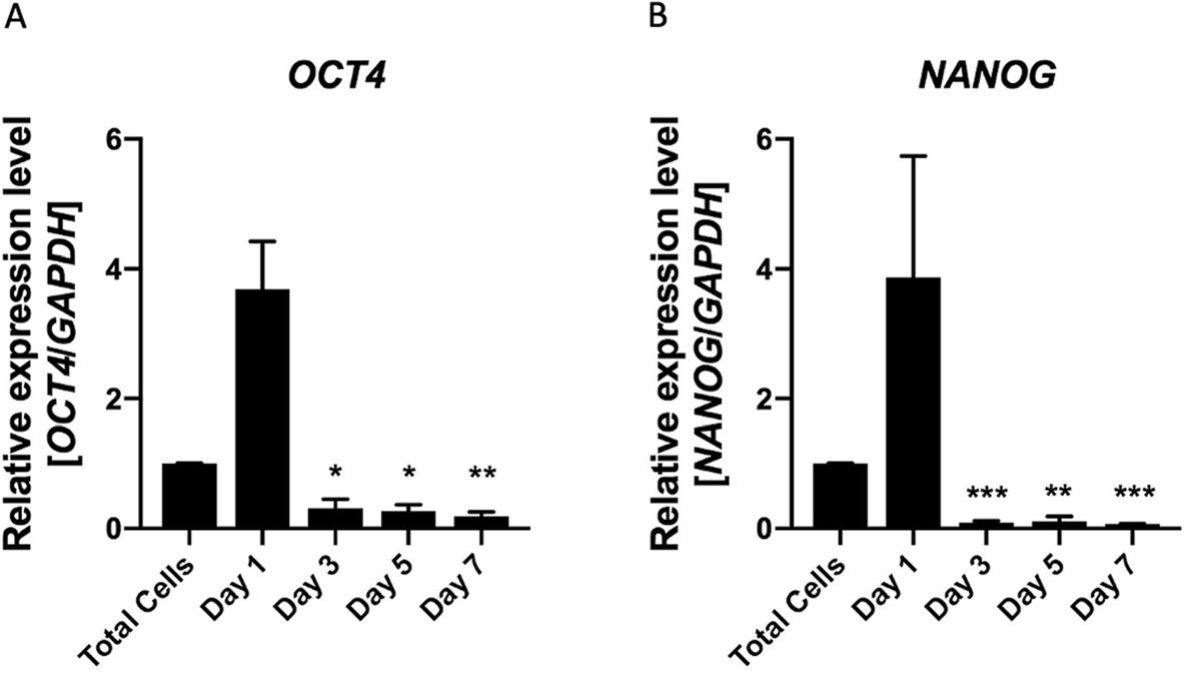
Cells isolated from the low-energy traumatized tissue express a robust stem cell phenotype immediately after isolation that is reduced over-time in monolayer culture conditions. Relative expression levels of **a** *OCT4* and **b** *NANOG* were investigated by q-RT-PCR on total cells (prior to the 2 h incubation for progenitor cells isolation) and isolated cells (adherent cells after the 2 h incubation) on days 1, 3, 5, and 7. Gene expression was normalized using *GAPDH* as an internal housekeeping control. Mean value + standard error of three independent donors. *p ≦ 0.05, **p ≦ 0.01, ***p ≦ 0.001, *T* test 2-tail

To investigate the multiprogenitor capacity of the cells isolated from low-energy trauma, we performed q-RT-PCR analysis for the genes *OCT4* and *NANOG*, whose expression and involvement in the regulation of stem cell properties have been previously reported in MSCs [18, 19]. The total cell fraction from low-energy traumatized tissue, prior to the 2 h incubation for progenitor cells isolation, was used as a reference for comparison. The progenitor cells were collected on days 1, 3, 5, and 7 following the tissue processing. Interestingly, low-energy progenitor cells expressed high levels of the stem cell markers *OCT4* (fold change [average + standard error]: total cells: 1.0 + 0.003, day 1: 3.7 + 0.7, *p* = 0.2; day 3: 0.3 + 0.1, *p* = 0.04; day 5: 0.3 + 0.1, *p* = 0.02; and day 7: 0.2 + 0.1, *p* = 0.01) and *NANOG* (fold change [average + standard error]: total cells: 1.0 + 0.003, day 1: 3.9 + 1.9, *p* = 0.4; day 3: 0.1 + 0.03, *p* = 0.001; day 5: 0.1 + 0.1, *p* = 0.01; and day 7: 0.05 + 0.02, *p* = 0.0005) 1 day after isolation, while expression of these genes was significantly reduced after 3 days in monolayer cell culture conditions (Fig. 2). Of note, although reduced, a low-level expression was detected after day 3 in culture. Altogether, these results demonstrate that progenitor cells isolated from the low-energy traumatized tissue express similar cell surface markers as previously reported MPCs isolated from high-energy trauma [12], while expressing a stem cell phenotype that decreases overtime, but it is not completely abrogated, in monolayer culture.

### Progenitor cells isolated from the low-energy traumatized muscle have the ability to differentiate into multiple mesenchymal lineages similar to progenitor cells from high-energy traumas

**Fig. 3:**
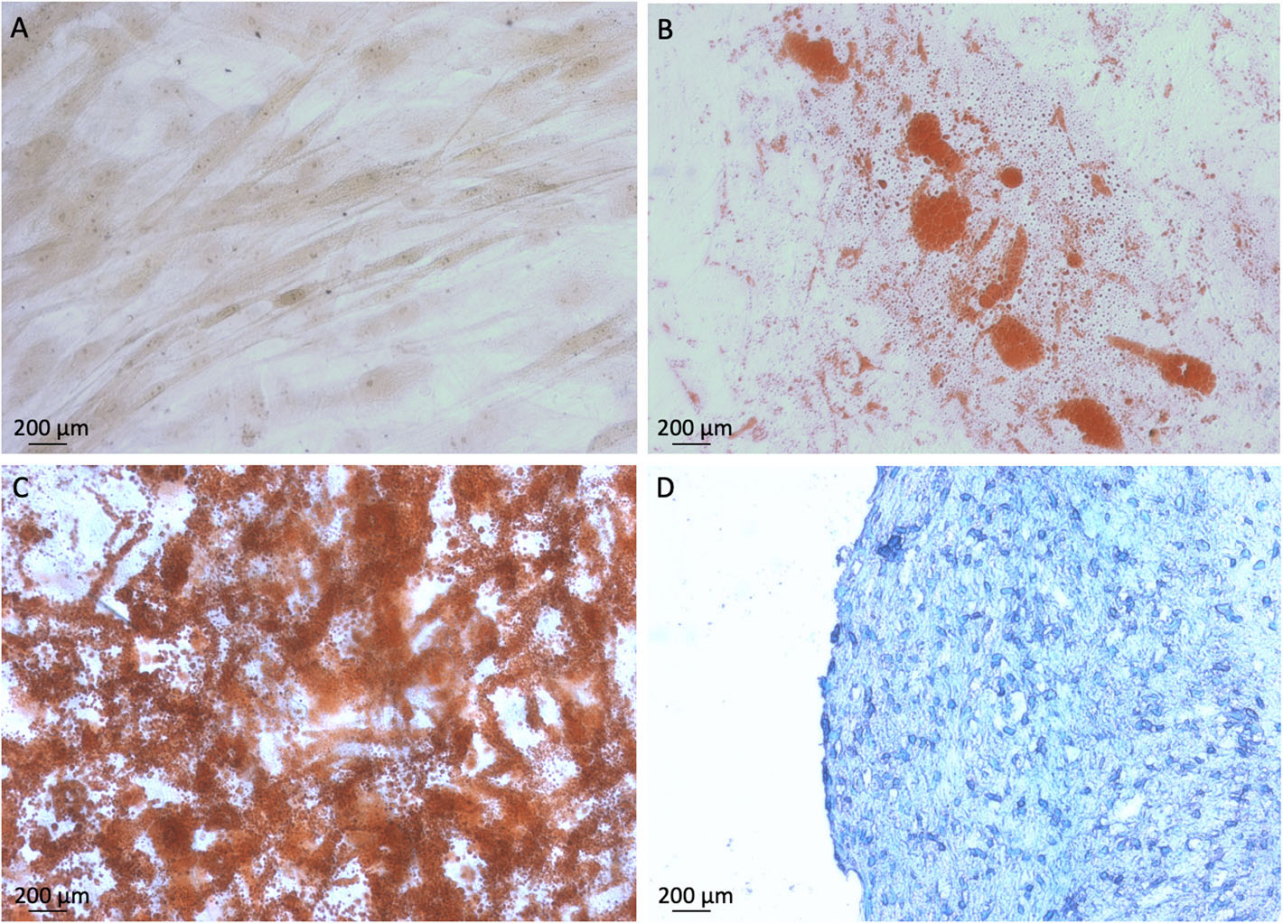
Progenitor cells isolated from the low-energy traumatized muscle have the ability to undergo adipogenic, osteogenic, and chondrogenic differentiation. Representative images are shown from **a** control cells compared to cells induced to differentiation for 4 weeks followed by **b** Oil Red O staining (adipogenic induction), **c** Alizarin red staining (osteogenic induction), and **d** Alcian blue staining (chondrogenic induction). Multiprogenitor cells cultured in adipogenic induction medium demonstrated intracellular lipid droplets that stained positively for Oil Red O. Multiprogenitor cells cultured in osteogenic induction medium exhibited enhanced mineralized matrix formation that stained positively for Alizarin red staining. Histological sections of the multiprogenitor cell pellet cultures showed a positive matrix staining with Alcian blue, demonstrating the presence of sulfated proteoglycan-rich extracellular matrix. Images are at ×20 magnification

To investigate the ability of the multiprogenitor cells from the low-energy traumatized tissue to undergo differentiation into the adipogenic, osteogenic, and chondrogenic pathways, cells were cultured in inductive media for each respective lineage differentiation. As previously reported for high-energy MPCs, monolayer culture conditions were used for osteogenesis and adipogenesis inductions, while a pellet culture condition was used for chondrogenesis induction [12]. After 4 weeks in adipogenic induction culture conditions, we observed intracellular lipid droplet accumulation by Oil Red O staining, which is consistent with an adipocyte phenotype compared to control cells cultured in growth medium (Fig. 3a, b). In addition, after 4 weeks in osteoinductive medium, we observed histological evidence of increased matrix mineralization by Alizarin red staining compared to control cells (Fig. 3a, c). Finally, after 4 weeks in chondrogenic induction culture conditions, histological sections of the cell pellet cultures were stained with Alcian blue, demonstrating the presence of a sulfated proteoglycan-rich extracellular matrix characteristic of cartilage tissue compared to control cells (Fig. 3a, d).

**Fig. 4:**
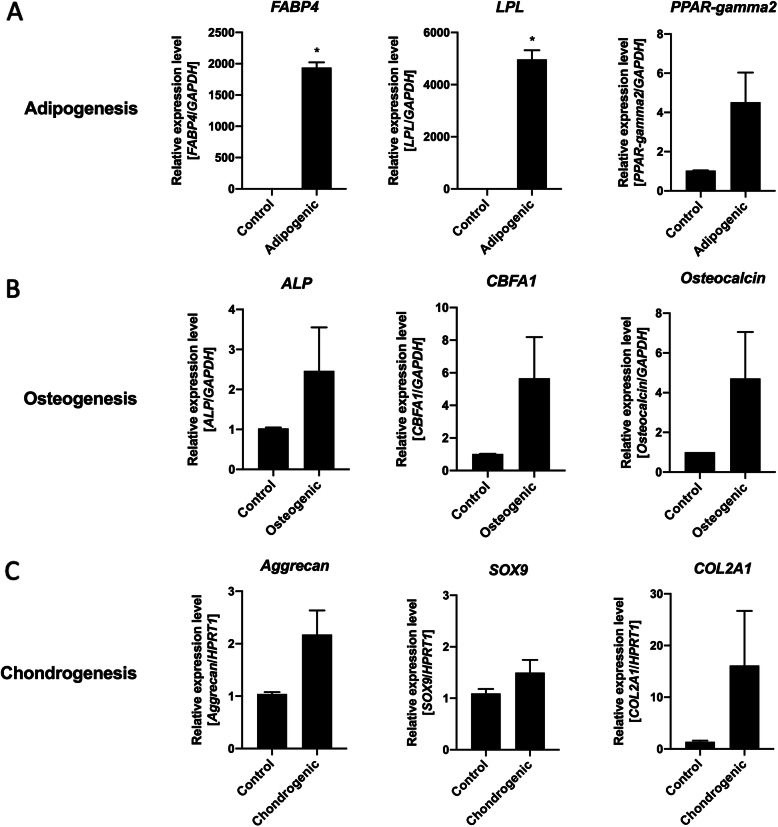
Progenitor cells isolated from the low-energy traumatized muscle express an adipogenic, osteogenic, and chondrogenic phenotype upon differentiation induction. Relative expression levels of **a** adipogenic genes (*FABP4*, *LPL*, and *PPAR-gamma2*), **b** osteogenic genes (*ALP*, *CBFA1*, and *Osteocalcin*), and **c** chondrogenic genes (*Aggrecan*, *SOX9*, and *COL2A1*) were investigated by q-RT-PCR on progenitor cells from the low-energy traumatized tissue following differentiation induction for 4 weeks. Gene expression was normalized using *GAPDH* or *HPRT1* as an internal housekeeping control as indicated. Mean value + standard error of three independent donors. *p ≦ 0.01, *T* test 2-tail

To confirm the ability of these cells to undergo differentiation, we performed gene expression analyses by q-RT-PCR targeting genes that are characteristic for each mesenchymal cell lineage (adipogenic: *FABP4*, *LPL*, and *PPAR-gamma2*; osteogenic: *ALP*, *CBFA1*, and *Osteocalcin*; and chondrogenic: *Aggrecan*, *SOX9*, and *COL2A1*). As shown on Fig. 4, all inductions resulted in increased expression of the lineage-specific genes compared to control cultures in growth medium (fold change [average + standard error]: *FABP4*: control: 1.0 + 0.001, adipogenic: 1938 + 84.2; *LPL*: control: 1.1 + 0.1, adipogenic: 4967 + 352; *PPAR-gamma2*: control: 1.0 + 0.02, adipogenic: 4.5 + 1.5; *ALP*: control: 1.0 + 0.02, osteogenic: 2.5 + 1.1; *CBFA1*: control: 1.0 + 0.01, osteogenic: 5.7 + 2.5; *Osteocalcin*: control: 1.0 + 0.001, osteogenic: 4.7 + 2.3; *Aggrecan*: control: 1.0 + 0.04, chondrogenic: 2.2 + 0.5; *SOX9*: control: 1.1 + 0.1, chondrogenic: 1.5 + 0.2; *COL2A1*: control: 1.4 + 0.2, chondrogenic: 16.1 + 10.5). These results demonstrate that following the induction, lineage-specific markers were demonstrated by each specific staining (Oil Red O, Alizarin red, and Alcian blue) and confirmed at the gene expression level by q-RT-PCR analyses for lineage-specific genes, which confirm the ability of the multiprogenitor cells isolated from the low-energy traumatized tissue to differentiate into the mesenchymal adipogenic, osteogenic, and chondrogenic lineages.

## Discussion

Adult multipotent progenitor cells are essential for the success of tissue engineering and regenerative medicine efforts. In this study, mesenchymal progenitor cells (MPCs) from the muscle tissue following low-energy trauma were isolated and cultured in vitro. This study is a continuation of our previous efforts to isolate and characterize MPCs from war-traumatized, military, high-energy blast injuries [12, 17]. Here, we expand and relate these initial findings to community, low-energy, civilian non-wartime blunt trauma. We believe this is a highly translational study that demonstrates that our previous discoveries found in a more limited, specialized patient population of war-traumatized service members are applicable to the general population and low-energy trauma patients. From the clinical and translational perspective, this manuscript fills a critical gap validating war-time findings and making them relevant to the civilian population. The MPCs isolated in this study from the low-energy tissue were adherent to the plastic culture dishes after 2 h of incubation, following similar isolation and culture conditions previously described for MPCs isolated from the severely traumatized muscle from high-energy injuries [12]. Low-energy MPCs were positive for CD73, CD90, and CD105, and negative for CD11b and CD45. The MPCs isolated from the low-energy traumatized muscle also demonstrated multipotential capacity, as upon induction with previously defined culture conditions [12], they differentiated into adipogenic, osteogenic, and chondrogenic lineages. Similar characteristics were previously reported for MPCs isolated and cultured from high-energy traumatized tissue samples [12]. Importantly, the multipotential capacity of low-energy traumatized MPCs was validated by robust and consistent lineage-specific expression of adipogenic, osteogenic, and chondrogenic genes. These results demonstrate that debrided muscle from low-energy trauma is a robust source of primary multiprogenitor cells with significant potential for translation into tissue regeneration clinical applications.

The isolation and characterization of progenitor cells in the low-energy traumatized muscle tissue that have similar characteristics to the progenitor cells isolated in high-energy traumatized tissue suggests that following either low- and high-energy injury, the cascade of events triggered as initial healing response are similar. By comparison, when the non-traumatized muscle is submitted to the same protocol for the isolation and characterization of MPCs, no cells are observed attached to the plate up to 2 weeks after plating and processing. As such, the non-traumatized (normal) muscle is not a suitable control for this study. Interestingly, the isolation of mesenchymal stem/stromal cells from the non-traumatized human muscle has been previously described [20]. However, in this study (i), a different cell harvesting protocol was used that most likely isolated different cell populations compared to the MPCs isolated here, and (ii) a distinct patient population diagnosed with osteoarthritis and undergoing routine total hip arthroplasty was recruited for tissue sampling and harvest [20]. Altogether, these differences make it challenging to establish a direct comparison between these cell populations and studies.

Importantly, while our study investigated a clinically relevant primary population of cells isolated from the site of injury, the exact origin of these cells is still unclear; it is possible that they comprise a quiescent resident population of cells in the non-traumatized muscle and/or a population of cells that migrate from the bone marrow to the site of injury in response to the wound-healing signaling events triggered by the trauma. Different origins of multipotential cell populations have been identified and characterized in a tissue-specific manner; the periosteum has been reported as the primary source of stem and/or progenitor cells that form the cartilage and bone during fracture repair [21]; bone marrow-derived MSCs have also been reported to selectively home to sites of injury, regardless of the tissue [22–24], and their migration signal(s) vary widely such as hypoxia, growth factors, and chemokines secreted by the injured cells and/or by immune cells also recruited to the site of injury [25–31]. Moreover, resident muscle stem (satellite) cells (known as MuSC) have been reported as major players during skeletal muscle regeneration, in combination with immune cells, fibroblasts, pericytes, and others [32, 33]. Human muscle-derived stem cells correspond to a population of early myogenic-committed progenitors with a perivascular/mesenchymal phenotypic signature that reside in the human skeletal muscle and display a high proliferation rate [33]. These cells differ from the MPCs as they are isolated from a population of poorly adhering cells between days 5 and 8 after plating from normal muscle samples [33], while the MPCs are isolated from a population of fast-adherent cells after 2 h of incubation under standard tissue-culture conditions (37 °C, 95% humidified air, and 5% CO_2_) [12] on the same day that the cells are isolated and plated from the low-energy traumatized muscle tissue. Additionally, a population of CD146+ cells that is anatomically and phenotypically distinct from satellite cells was isolated from the interstitium of the normal adult human skeletal muscle tissue [34]. These cells also differ from the population of MPCs isolated in this study as single-cell suspensions were seeded for non-clonal or multi-clonal cultures without the 2h adherence incubation step [34] used here and that we previously described [12]. As such, distinct populations of multiprogenitor cells are most likely been isolated and investigated in each of these studies.

Both low- and high-energy trauma will result in structural tissue-damage, impaired tissue perfusion and trigger the activation of an inflammatory response [35]. As a result, we hypothesize that the recruitment, induction, and proliferation of stem and/or multiprogenitor cells are an essential component of the wound-healing response process. Nonetheless, inhibition and/or pathological inflammatory response to injury can impair stem cell function and lead to unsatisfactory tissue regeneration and poor recovery outcomes [35]. As such, Torossian et al. [36] recently demonstrated that an increase in secreted Oncostatin M produced by activated macrophages—a cytokine known to promote osteogenic differentiation in mesenchymal stromal cells—also promoted osteogenic differentiation and mineralization of muscle-derived stromal cells surrounding the site where neurogenic heterotopic ossification developed. Furthermore, fibrosis is a well-known pathologic event of the normal wound healing process that leads to suboptimal tissue regeneration, in particular limiting the functional regeneration of the musculoskeletal tissues. It is likely that in high-energy trauma, the exacerbated inflammatory response, generated as part of the wound healing and tissue regeneration process, leads to an undesirable repair response, resulting in the formation of fibrotic tissue that compromises efficient wound healing and tissue regeneration [37, 38]. Instead, low-energy trauma most likely triggers a less exacerbated inflammatory response, promoting less undesirable fibrotic tissue formation and more tissue repair and regeneration.

## Conclusions

In summary, we have isolated and characterized a population of primary mesenchymal progenitor cells harvested from the debrided human muscle tissue following low-energy community trauma. Upon induction with previously defined culture conditions [12], these progenitor cells were capable of differentiating into multiple mesenchymal lineages and have the potential to play a role in future tissue engineering and regenerative medicine efforts.

## Supplementary Information

**Additional file 1: Supplemental Table 1.** Primer sequences (5′ to 3′) and TaqMan assays used for relative gene-expression analysis by quantitative reverse transcription polymerase chain reaction (q-RT-PCR).

## Data Availability

All data generated or analyzed during this study are included in this published article.

## Abbreviations

APC: Allophycocyanin fluorescence
BV421: Brilliant Violet 421 fluorescence
°C: Degrees Celsius
CD73 +: Cluster of differentiation 73 positive
CD90 +: Cluster of differentiation 90 positive
CD105 +: Cluster of differentiation 105 positive
cDNA: Complementary deoxyribonucleic acid
Cy7: Cyanine7 fluorescence
FBS: Fetal bovine serum
FITC: Fluorescein isothiocyanate fluorescence
HBSS: Hank’s balanced salt solution
ITS: Insulin, human transferrin, and sodium selenite
MPCs: Mesenchymal progenitor cells
μM: Micromolar
MuSC: (Resident) Muscle stem (satellite) cells
PE: Phycoerythrin fluorescence
q-RT-PCR: Real-time quantitative reverse transcription polymerase chain reaction
RNA: Ribonucleic acid

## References

[1] Azad A, Kang HP, Alluri RK, Vakhshori V, Kay HF, Ghiassi A (2019). Epidemiological and treatment trends of distal radius fractures across multiple age groups. J Wrist Surg.

[2] Cummings SR, Kelsey JL, Nevitt MC, O'Dowd KJ (1985). Epidemiology of osteoporosis and osteoporotic fractures. Epidemiol Rev.

[3] Diamantopoulos AP, Rohde G, Johnsrud I, Skoie IM, Hochberg M, Haugeberg G (2012). The epidemiology of low- and high-energy distal radius fracture in middle-aged and elderly men and women in Southern Norway. Plos One.

[4] Hove LM, Fjeldsgaard K, Reitan R, Skjeie R, Sörensen FK (1995). Fractures of the distal radius in a Norwegian city. Scand J Plast Reconstr Surg Hand Surg.

[5] MacIntyre NJ, Dewan N (2016). Epidemiology of distal radius fractures and factors predicting risk and prognosis. J Hand Ther.

[6] Marsh DR, Li G (1999). The biology of fracture healing: optimising outcome. Br Med Bull.

[7] Xie Y, Zhang L, Xiong Q, Gao Y, Ge W, Tang P (2019). Bench-to-bedside strategies for osteoporotic fracture: from osteoimmunology to mechanosensation. Bone Res.

[8] Berebichez-Fridman R, Montero-Olvera PR (2018). Sources and clinical applications of mesenchymal stem cells: state-of-the-art review. Sultan Qaboos Univ Med J.

[9] Boquest AC, Noer A, Collas P (2006). Epigenetic programming of mesenchymal stem cells from human adipose tissue. Stem Cell Rev.

[10] Caterson EJ, Nesti LJ, Danielson KG, Tuan RS (2002). Human marrow-derived mesenchymal progenitor cells: isolation, culture expansion, and analysis of differentiation. Mol Biotechnol.

[11] Flynn A, Barry F, O'Brien T (2007). UC blood-derived mesenchymal stromal cells: an overview. Cytotherapy..

[12] Nesti LJ, Jackson WM, Shanti RM, Koehler SM, Aragon AB, Bailey JR (2008). Differentiation potential of multipotent progenitor cells derived from war-traumatized muscle tissue. J Bone Joint Surg Am.

[13] Nöth U, Osyczka AM, Tuli R, Hickok NJ, Danielson KG, Tuan RS (2002). Multilineage mesenchymal differentiation potential of human trabecular bone-derived cells. J Orthop Res.

[14] Zheng C, Chen J, Liu S, Jin Y (2019). Stem cell-based bone and dental regeneration: a view of microenvironmental modulation. Int J Oral Sci.

[15] Jackson WM, Alexander PG, Bulken-Hoover JD, Vogler JA, Ji Y, McKay P (2013). Mesenchymal progenitor cells derived from traumatized muscle enhance neurite growth. J Tissue Eng Regen Med.

[16] Zupanc HRH, Alexander PG, Tuan RS (2017). Neurotrophic support by traumatized muscle-derived multipotent progenitor cells: role of endothelial cells and vascular endothelial growth factor-A. Stem Cell Res Ther.

[17] Woodard GE, Ji Y, Christopherson GT, Wolcott KM, Hall DJ, Jackson WM (2014). Characterization of discrete subpopulations of progenitor cells in traumatic human extremity wounds. PLoS One.

[18] Greco SJ, Liu K, Rameshwar P (2007). Functional similarities among genes regulated by OCT4 in human mesenchymal and embryonic stem cells. Stem Cells.

[19] Matic I, Antunovic M, Brkic S, Josipovic P, Mihalic KC, Karlak I (2016). Expression of OCT-4 and SOX-2 in bone marrow-derived human mesenchymal stem cells during osteogenic differentiation. Open Access. Maced J Med Sci.

[20] Čamernik K, Mihelič A, Mihalič R, Marolt Presen D, Janež A, Trebše R (2019). Skeletal-muscle-derived mesenchymal stem/stromal cells from patients with osteoarthritis show superior biological properties compared to bone-derived cells. Stem Cell Res.

[21] Bragdon BC, Bahney CS (2018). Origin of reparative stem cells in fracture healing. Curr Osteoporos Rep.

[22] Ortiz LA, Gambelli F, McBride C, Gaupp D, Baddoo M, Kaminski N (2003). Mesenchymal stem cell engraftment in lung is enhanced in response to bleomycin exposure and ameliorates its fibrotic effects. Proc Natl Acad Sci U S A.

[23] Liu Y, Yan X, Sun Z, Chen B, Han Q, Li J (2007). Flk-1+ adipose-derived mesenchymal stem cells differentiate into skeletal muscle satellite cells and ameliorate muscular dystrophy in mdx mice. Stem Cells Dev.

[24] Ferrari G, Cusella-De Angelis G, Coletta M, Paolucci E, Stornaiuolo A, Cossu G (1998). Muscle regeneration by bone marrow-derived myogenic progenitors. Science..

[25] Spaeth E, Klopp A, Dembinski J, Andreeff M, Marini F (2008). Inflammation and tumor microenvironments: defining the migratory itinerary of mesenchymal stem cells. Gene Ther.

[26] Yagi H, Soto-Gutierrez A, Parekkadan B, Kitagawa Y, Tompkins RG, Kobayashi N (2010). Mesenchymal stem cells: mechanisms of immunomodulation and homing. Cell Transplant.

[27] Granero-Moltó F, Weis JA, Miga MI, Landis B, Myers TJ, O'Rear L (2009). Regenerative effects of transplanted mesenchymal stem cells in fracture healing. Stem Cells.

[28] Cook KM, Sifri ZC, Baranski GM, Mohr AM, Livingston DH (2013). The role of plasma granulocyte colony stimulating factor and bone marrow dysfunction after severe trauma. J Am Coll Surg.

[29] Li L, Jiang J (2011). Regulatory factors of mesenchymal stem cell migration into injured tissues and their signal transduction mechanisms. Front Med.

[30] Busletta C, Novo E, Valfrè Di Bonzo L, Povero D, Paternostro C, Ievolella M (2011). Dissection of the biphasic nature of hypoxia-induced motogenic action in bone marrow-derived human mesenchymal stem cells. Stem Cells.

[31] Naaldijk Y, Johnson AA, Ishak S, Meisel HJ, Hohaus C, Stolzing A (2015). Migrational changes of mesenchymal stem cells in response to cytokines, growth factors, hypoxia, and aging. Exp Cell Res.

[32] Magadum A, Engel FB (2018). PPARβ/δ: Linking Metabolism to Regeneration. Int J Mol Sci..

[33] Lorant J, Saury C, Schleder C, Robriquet F, Lieubeau B, Négroni E (2018). Skeletal muscle regenerative potential of human MuStem cells following transplantation into injured mice muscle. Mol Ther.

[34] Persichini T, Funari A, Colasanti M, Sacchetti B (2017). Clonogenic, myogenic progenitors expressing MCAM/CD146 are incorporated as adventitial reticular cells in the microvascular compartment of human post-natal skeletal muscle. Plos One.

[35] Thurairajah K, Broadhead ML, Balogh ZJ (2017). Trauma and stem cells: biology and potential therapeutic implications. Int J Mol Sci..

[36] Torossian F, Guerton B, Anginot A, Alexander KA, Desterke C, Soave S (2017). Macrophage-derived oncostatin M contributes to human and mouse neurogenic heterotopic ossifications. JCI Insight..

[37] Forsberg JA, Pepek JM, Wagner S, Wilson K, Flint J, Andersen RC (2009). Heterotopic ossification in high-energy wartime extremity injuries: prevalence and risk factors. J Bone Joint Surg Am.

[38] Potter BK, Burns TC, Lacap AP, Granville RR, Gajewski DA (2007). Heterotopic ossification following traumatic and combat-related amputations. Prevalence, risk factors, and preliminary results of excision. J Bone Joint Surg Am.

