# Supplementary material for: Characterization of traumatized muscle-derived multipotent progenitor cells from low-energy trauma
Source: Stem Cell Res Ther. 2021 Jan 6;12:6. doi: 10.1186/s13287-020-02038-2 (PMC7788846; doi:10.1186/s13287-020-02038-2)
Supplement: Supplementary file 1 — Additional file 1: Supplemental Table 1. Primer sequences (5′ to 3′) and TaqMan assays used for relative gene-expression analysis by quantitative reverse transcription polymerase chain reaction (q-RT-PCR). [file 13287_2020_2038_MOESM1_ESM.docx]

**Supplemental Table 1 –** Primer sequences (5’ to 3’) and TaqMan assays used for relative gene-expression analysis by quantitative Reverse Transcription Polymerase Chain Reaction (q-RT-PCR).

| **Gene** | **Primer Sequence (5’ to 3’)** |
| --- | --- |
| *FABP4*-Forward | GGCCAGGAATTTGACGAAGT |
| *FABP4*-Reverse | TCAACGTCCCTTGGCTTATG |
| *LPL*-Forward | GAGATTTCTCTGTATGGCACCGT |
| *LPL*-Reverse | TCTGCAAATGAGACACTTTCTCC |
| *PPAR-gamma2*-Forward | TGAATGTGAAGCCCATTGAAG |
| *PPAR-gamma2-*Reverse | TGCAGTAGCTGCACGTGTTC |
| *ALP*-Forward | CAGAAGCTCAACACCAACGTG |
| *ALP*-Reverse | GTCAGGGACCTGGGCATT |
| *CBFA1*-Forward | CCGAGCTACGAAATGCCTCT |
| *CBFA1*-Reverse | TGAAACTCTTGCCTCGTCCG |
| *Osteocalcin*-Forward | GCCTTTGTGTCCAAGCAGG |
| *Osteocalcin*-Reverse | TCCTGAAAGCCGATGTGGT |
| *Aggrecan*-Forward | CGGGTCAACAGTGCCTATCA |
| *Aggrecan*-Reverse | ACGATGCCTTTCACCACGA |
| *SOX9*-Forward | ACGCACATCTCCCCCAAC |
| *SOX9*-Reverse | TCGCTTCAGGTCAGCCTTG |
| *COL2A1*-Forward | GGCAATAGCAGGTTCACGTACA |
| *COL2A1*-Reverse | CGATAACAGTCTTGCCCCACTT |
| *GAPDH*-Forward | ACAGTCCATGCCATCACTGC |
| *GAPDH*-Reverse | GCCTGCTTCACCACCTTCTT |
| *HPRT1*-Forward | GAGGAGTCCTGTTGATGTTGCCAG |
| *HPRT1*-Reverse | GGCTGGCCTATAGGCTCATAGTGC |

| **Gene** | **Probe Sequence Information/Assay ID (ThermoFisher Scientific)** |
| --- | --- |
| *OCT4* | Hs04260367_g1 |
| *NANOG* | Hs04260366_g1 |
| *GAPDH* | Hs02786624_g1 |
